# Probiotic *Bifidobacterium breve* in Improving Cognitive Functions of Older Adults with Suspected Mild Cognitive Impairment: A Randomized, Double-Blind, Placebo-Controlled Trial

**DOI:** 10.3233/JAD-200488

**Published:** 2020-09-01

**Authors:** Jinzhong Xiao, Noriko Katsumata, Francois Bernier, Kazuya Ohno, Yuki Yamauchi, Toshitaka Odamaki, Kenji Yoshikawa, Kumie Ito, Toshiyuki Kaneko

**Affiliations:** a Morinaga Milk Industry Co., Ltd., Next Generation Science Institute, Kanagawa, Japan; b Huma R & D Co. Ltd, Tokyo, Japan; c Nihonbashi Sakura Clinic, Tokyo, Japan; d Tokyo Skytree Station Medical Clinic, Tokyo, Japan

**Keywords:** *Bifidobacterium*, clinical trial, dementia, memory, mild cognitive impairment, probiotics

## Abstract

**Background:**

Probiotics use has been associated with modulation of inflammation and considered as a possible intervention for CNS diseases such as mild cognitive impairment (MCI) and dementia.

**Objective:**

We aimed to test the effect of the probiotic strain, *Bifidobacterium breve* A1 (MCC1274), to restore cognition in a physically healthy, suspected MCI population.

**Methods:**

In this randomized, double-blind, placebo-controlled trial, 80 healthy older adults suffering from MCI were divided into two even groups to receive once daily either probiotic (*B. breve* A1, 2×10^10^ CFU) or placebo for 16 weeks using a computer-generated algorithm. Cognitive functions were assessed by the Repeatable Battery for the Assessment of Neuropsychological Status (RBANS) and the Japanese version of the MCI Screen (JMCIS) tests before and after the study as primary and secondary endpoints, respectively.

**Results:**

79 participants completed the study, and no adverse events were observed. RBANS total score was significantly improved in probiotic group compared with placebo (mean between-group difference 11.3 [95% CI 6.7 to 15.8]; *p* < 0.0001) after 16 weeks of consumption, in particular with significant improvement in domain scores of immediate memory, visuospatial/constructional, and delayed memory (*p* < 0.0001), in both intention-to-treat (ITT) analysis and per-protocol (PP) analysis. JMCIS score was also improved versus placebo in ITT analysis (*p* = 0.052) and PP analysis (*p* = 0.036).

**Conclusion:**

Study results indicate *B. breve* A1 is a safe and effective approach for improving memory functions of suspected MCI subjects.

## INTRODUCTION

Mild cognitive impairment (MCI) is characterized by a decline of cognitive functions that does not usually interfere with activities of daily living but is associated with the risk of developing sporadic Alzheimer’s disease (AD) or other dementia within a few years if left untreated [1]. Numerous studies were, therefore, conducted to estimate the prevalence of MCI in the older adult population. According to the expanded Mayo Clinic criteria, the average prevalence of MCI varies between 7–42% depending on the studied population, reaching an average frequency of 18.9% amongst >65 years old [2].

The number of AD cases is now reaching epidemic proportions with an estimated one new patient being diagnosed every 65 seconds according to the Alzheimer’s Association web site with an estimated 135 million cases worldwide by the year 2050 (https://www.alz.org/alzheimers-dementia/facts-figures), causing an unprecedented burden to society. Current AD medications only have temporary beneficial symptomatic effects and are not effective in alleviating any symptoms of MCI [3]. Moreover, recent clinical trials in both early sporadic and familial AD patients using anti-amyloid-*β* strategies to block or clear various form of amyloid-*β* (the proposed cause of AD) all fell short of expectations [4] (DIAN study, https://www.clinicaltrialsarena.com/comment/dian-tu-drug-trial/), while less than a handful of anti-amyloid options are still being pursued. As a result, novel promising strategies for early dementia prevention are being uncovered. Recent studies, for example, suggested that MCI and AD could be due to peripheral and central mild polymicrobial infections as the source of brain inflammation, brain atrophy, neuronal cell death, and AD hallmarks such as amyloid plaques and tangles [5, 6]. Other emerging strategies are also looking at changes in lifestyle and nutrition as possible practical approaches to treat or prevent dementia [7, 8]. The very recent findings that dietary interventions such as oligomannates and ketogenic medium-chain triglyceride consumption resulted in cognition amelioration in AD and/or MCI point at the role of gut dysbiosis-promoted neuroinflammation in dementia and at the importance of considering the remodeling of the gut microbiota as novel therapies [9, 10].

Probiotics are live microorganisms that can provide health benefits when consumed in regular amounts [11]. Probiotics have been proposed to influence the CNS, and their use is now under active investigations for treating neurological disorders [12]. These investigations have been prompted from observations that gut microbiota in those disorders patients is different from those of healthy individuals and by the discoveries that several probiotics possess anti-inflammatory and modulatory effects on our immune system, which plays an important role in neuropsychological and neurodegenerative diseases [13]. In fact, gut microbes can have positive or negative effects on the brain environment using various known bidirectional pathways of communication, including hepatic and gallbladder metabolism, immune-modulatory responses, neuronal innervation (vagal nerve), enteroendocrine, and microbial metabolite signaling (gut-brain axis) [12].

In that context, our previous studies evaluating a probiotic strain, *Bifidobacterium breve* A1, in an AD mouse model is relevant since it demonstrated the therapeutic potential of this probiotic in managing memory functions as well as suppressing inflammation and immune-reactive genes that are induced by amyloid-*β* accumulation in brain [14]. We also previously conducted a randomized, double-blind, placebo-controlled pilot trial evaluating *B. breve* A1 in human subjects with self-reported memory complaints [15]. Although the trial did not meet its primary endpoint, we did observe significant improvement of immediate memory in the subgroup analysis of MCI subjects with lower memory scores at baseline compared to placebo when assessed by Repeatable Battery for the Assessment of Neuropsychological Status (RBANS), which prompted us to conduct further investigations.

The aim of this present study was, therefore, to test the hypothesis that *B. breve* A1 can ameliorate cognitive functions in a physiological healthy, suspected MCI population by conducting another double-blind placebo-controlled trial.

## MATERIALS AND METHODS

This study was a double-blind, randomized placebo-controlled trial to study the effect of *B. breve* A1 in subjects with suspected MCI who had lower RBANS total score and is registered at University Hospital Medical Research Network, number UMIN000037725. Such patient population was selected because *B. breve* A1 consumption seems more effective in subjects with more cognitive deficits as observed in our previous study [15].

### Participants

Older adults aged 50–79 years living in the Tokyo Metropolitan area were recruited from the volunteer bank of a clinical research organization (Huma R & D Co. Ltd, Tokyo, Japan). All subjects underwent a medical examination and Mini-Mental State Examination (MMSE) (Visit 1). Subjects were selected according to the following inclusion criteria 1) age 50–79 years with Japanese nationality, 2) MMSE score 22 or more. Exclusion criteria were as follows: 1) diagnosed with dementia, 2) under exercise therapy or dietetic therapy, 3) allergy for test food, 4) history of either medicine or alcohol dependence syndrome, 5) history of mental illness (depression) or sleep disturbance, 6) on a night-shift or is a shift worker, 7) lifestyle is hugely irregular, 8) an unbalanced diet, 9) being treated or history of serious diseases such as diabetes, liver disease (hepatitis), renal disease, heart disease, thyroid disease, adrenal disease, or other serious metabolic disease, 10) use of health foods, supplements, and medicines that may affect cognitive function, 11) history of significant surgery involving gastrectomy, gastrointestinal surgery, bowel resection, and digestive system (excluding appendicitis), 12) participation in other clinical studies within the past 3 months or who is planning to participate in other clinical studies during the current study, 13) blood draw of 200 mL within the past one month or 400 mL within the past three months, 14) under planning to get pregnant after the day of informed consent or is currently pregnant and lactating, 15) cannot keep the daily records, 16) judged ineligible based on the screening data or by the study physician.

**Fig.1:**
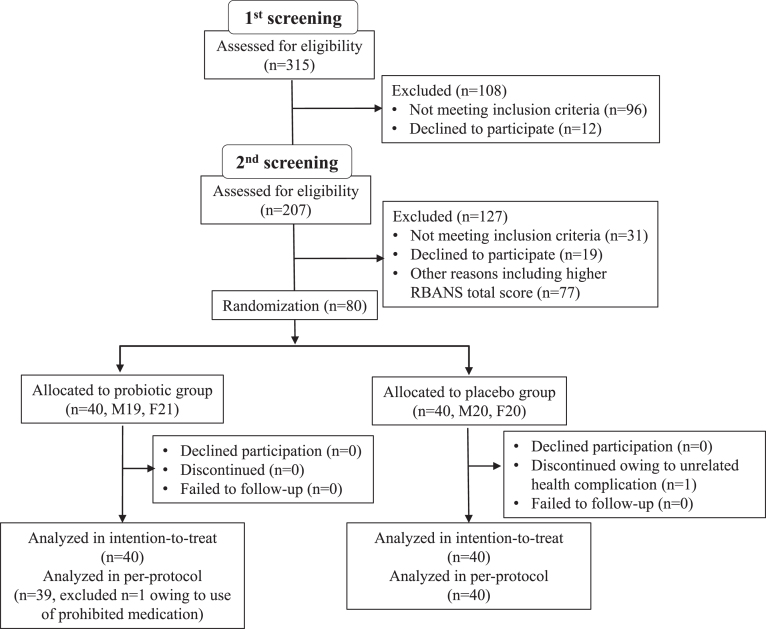
Trial profile.

Participants’ characteristics are shown in Table 1. Subjects were recruited between June to August 2019, in Nihonbashi Sakura Clinic, Tokyo, Japan and Tokyo Skytree Station Medical Clinic, Tokyo, Japan. In total, 315 subjects were screened (visit 1, –10 to –6 week), and 207 met the study criteria (Fig. 1). All selected subjects came for a second examination (visit 2, –6 to –2 week) for baseline RBANS score determination before randomization at the subsequent visit (visit 3, week 0). At the second screening, subjects were further selected for those with lower RBANS total score. A total of 80 subjects (age (years) 61.1±7.2, 50–76; MMSE 24.5±1.6, 22–28; RBANS total scores 31.5±9.2, 11–45) were enrolled in the randomization. This study was done with the approval of the ethical committee of Nihonbashi Egawa Clinic, and was based on the tenets of Declaration of Helsinki. Informed consent was obtained from each subject.

**Table 1:** Participants characteristics at baseline

	Probiotic (*n* = 40)	placebo (*n* = 40)
Age (y)	61.3 (7.7)	60.9 (6.9)
Sex (M/F)	19/21	20/20
Body weight (kg)	56.2 (8.5)	58.0 (8.5)
Height (cm)	163.2 (8.8)	163.2 (9.3)
Body mass index (kg · m^–2^)	21.0 (2.1)	21.7 (1.9)
Systolic blood pressure (mmHg)	124.0 (14.2)	120.9 (12.8)
Diastolic blood pressure (mmHg)	71.4 (8.7)	72.5 (9.5)
Heart rate (bpm)	68.4 (8.4)	70.0 (11.6)

Data are number or mean (SD).

### Intervention

We prepared capsules containing lyophilized powder of *B. breve* A1 (stocked as MCC1274 in Morinaga Culture Collection, Zama, Japan), a strain originating from an infant, which mainly contained maize starch as a carrier. We confirmed that each capsule contained 1×10^10^ CFU or more using a microbial colony counting methodology. Placebo capsules were composed of maize starch only and were identical in appearance, weight, and smell. Each participant was asked to consume two probiotic or placebo capsules daily for 16 weeks.

For the primary outcome, the Japanese version of RBANS test was administered by clinicians to assess multiple cognitive domains. It includes 12 standard cognitive subtests grouped in five domains: immediate memory (list learning and story memory), visuospatial/constructional (figure copy and line orientation), language (picture naming and semantic fluency), attention (digit span and digit symbol coding), and delayed memory (list recall, list recognition, story recall, and figure recall). A different version was used for each examination to avoid the effect of learning. The Japanese version of the MCI Screen (JMCIS) score was also added in this study as a secondary endpoint. It is based on a 10-word recall test and is considered to be the most sensitive test for discriminating between healthy aging and MCI[16]. Medical interviews and blood tests were performed at screening (visit 1) and at the end of the trial (visit 4, week 16). Body weight, blood pressure, and heart rate were measured at each visit.

### Randomization and masking

Eligible participants were distributed in a 1 : 1 ratio to the two groups (probiotic or placebo) of the study, according to a computer-generated random sequence. Any member of the research team did not know the allocated sequence until the study end and database lock. Given the safety record of *B. breve* A1, no provision for emergency unmasking of study participants was considered, and therefore, no allocation sequence copies were held at the recruitment sites.

### Statistical analysis

In our previous pilot trial, we observed significant improvement in immediate memory compared to placebo in the subgroup analysis of subjects with lower memory scores at baseline (probiotic group: *n* = 27, placebo group: *n* = 17) [15]. A sample size of 80 participants (40 per each group) was enrolled to confirm the previous study as well as to look at the effect on other memory parameters by *B. breve* A1 consumption. This sample size was expected to achieve 80% power to detect 6-point difference change from baseline of RBANS score between the probiotic and placebo groups, assuming a standard deviation of 9 in both groups, a two-side test with *α*= 0.05, and an attrition rate of 10%.

A total of 39 placebos and 40 treated participants completed the final examination. One female in the placebo group left the study at week 14 due to unrelated health complications, and one male in the probiotic arm started taking statins from week 7 during the study. Once data collection was completed, all data were fixed before the code-breaking. The main analysis for all primary and secondary outcome measures was done on the intention-to-treat (ITT) with complete-case analysis for missing data. Per-protocol (PP) analysis by excluding data of the subject in probiotic arm who took prohibited medication was applied for the primary and secondary outcomes. Statistical analysis was performed using SPSS software version 26 (IBM, Tokyo, Japan) with significance set at *p* < 0.05. After examining the value distribution to be normal, we used Analysis of Co-Variance (ANCOVA) model to compare the values at week 16 for the probiotic group versus the placebo group of RBANS score and JMCIS score with baseline score as a covariate. The Student’s *t*-test was used for comparing the changes of means. Safety of *B. breve* A1 was assessed on all participants by comparing any changes with Student’s *t*-test.

## RESULTS

Out of 80 final study participants, 79 subjects completed the study. The rate of consumption of the supplements for 16 weeks was considerably high in the 79 participants (>99.9%). Baseline characteristics of the participants were pretty much identical, and there were no significant baseline differences between groups (Table 1). Besides having mild cognitive function impairment, all participants were physically healthy, non-obese, older adults with no blood pressure abnormalities.

### Cognitive function primary outcomes

**Fig.2:**
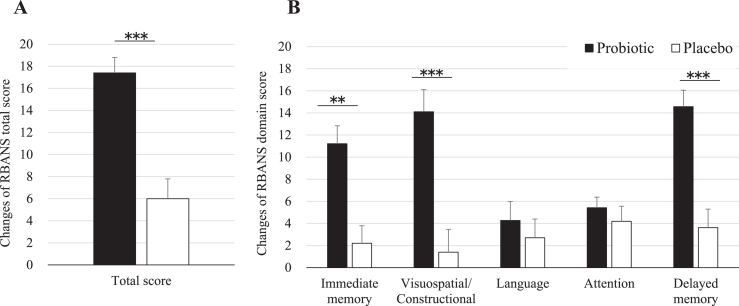
Changes of RBANS scores at 16 weeks from baseline. Values are indicated as mean with error bars as SE. **p < 0.001, ***p < 0.0001, inter-group difference, Student’s *t*-test. RBANS, Repeatable Battery for the Assessment of Neuropsychological Status.

Table 2 shows the results of the neuropsychological tests from baseline to 16 weeks after probiotic or placebo consumption as for ITT analysis. RBANS total score was significantly improved by 11.3 points by *B. breve* A1 (95% CI 6.7 to 15.8, *p* < 0.0001). Each memory domain score was analyzed. As shown in Table 2, compared to placebo group, immediate memory, visuospatial/constructional score, and delayed memory were improved by 9.2 points (95% CI 5.1 to 13.3, *p* < 0.0001), 11.4 points (95% CI 6.8 to 16.0, *p* < 0.0001), and 11.0 points (95% CI 6.6 to 15.3, *p* < 0.0001), respectively, by *B. breve* A1 consumption. Language shows a trend of improvement over placebo in the probiotic group (95% CI –0.2 to 7.2, *p* = 0.064). For the attention parameter, no improvement was observed after consumption (95% CI –2.7 to 3.7, *p* = 0.74). RBANS results as for PP analysis were very similar to ITT analysis (Table 3). Figure 2 shows the changes of RBANS scores at 16 weeks from baseline. Significant inter-group difference was observed in RBANS total scores and the domain scores of visuospatial/constructional and delayed memory and immediate memory (Fig. 2A, B). The secondary outcome (JMCIS) was also improved in the probiotic group over placebo in ITT analysis (*p* = 0.052, Table 2) and PP analysis (*p* = 0.036, Table 3).

**Table 2:** Results of the neuropsychological tests (ITT)

	Baseline		16 weeks	
	Placebo	Probiotic	Placebo	Probiotic	Difference (95% CI)	*p*
RBANS total score	32.4 (7.5)	30.5 (10.6)	38.3 (13.0)	47.9 (13.4)	11.3 (6.7 to 15.8)	<0.0001
Immediate memory	36.4 (8.4)	36.9 (10.5)	38.7 (9.9)	48.2 (11.2)	9.2 (5.1 to 13.3)	<0.0001
Visuospatial/Constructional	34.4 (14.4)	32.1 (13.2)	35.8 (13.5)	46.2 (10.0)	11.4 (6.8 to 16.0)	<0.0001
Language	47.3 (7.8)	49.9 (10.7)	50.1 (8.8)	54.2 (8.1)	3.5 (–0.2 to 7.2)	0.064
Attention	49.2 (10.0)	45.7 (11.0)	53.3 (11.8)	51.1 (10.2)	0.5 (–2.7 to 3.8)	0.74
Delayed memory	31.1 (12.3)	31.1 (12.0)	34.6 (13.5)	45.6 (14.2)	11.0 (6.6 to 15.3)	<0.0001
JMCIS score	63.2 (7.5)	61.3 (9.2)	60.5 (9.9)	62.6 (8.4)	3.5 (0.2 to 6.9)	0.052

Baseline (Placebo: *n* = 40, Probiotic: *n* = 40), 16 weeks (Placebo: *n* = 39, Probiotic: *n* = 40). Values are indicated as mean (SD). Differences are indicated by changes of LSM between Placebo and Probiotice at 16 weeks. Effect of Probiotic was indicated in intergroup difference (95% CI) and p value by ANCOVA in intention-to-treat (ITT) analysis. RBANS, Repeatable Battery for the Assessment of Neuropsychological Status; JMCIS, The Japanese version of the MCI Screen.

**Table 3:** Results of the neuropsychological tests (PP)

	Baseline		16 weeks	
	Placebo	Probiotic	Placebo	Probiotic	Difference (95% CI)	*p*
RBANS total score	32.4 (7.5)	30.4 (10.7)	38.3 (13.0)	48.0 (13.6)	11.5 (6.9 to 16.1)	<0.0001
Immediate memory	36.4 (8.4)	36.9 (10.6)	38.7 (9.9)	48.5 (11.2)	9.5 (5.4 to 13.6)	<0.0001
Visuospatial/Constructional	34.4 (14.4)	32.0 (13.4)	35.8 (13.5)	46.0 (10.1)	11.3 (6.6 to 15.9)	<0.0001
Language	47.3 (7.8)	49.8 (10.8)	50.1 (8.8)	53.9 (8.0)	3.2 (–0.5 to 6.9)	0.085
Attention	49.2 (10.0)	45.6 (11.1)	53.3 (11.8)	51.1 (10.4)	0.7 (–2.6 to 4.0)	0.67
Delayed memory	31.1 (12.3)	31.3 (12.0)	34.6 (13.5)	45.9 (14.3)	11.1 (6.6 to 15.5)	<0.0001
JMCIS score	63.2 (7.5)	61.4 (9.3)	60.5 (9.9)	63.0 (8.2)	3.5 (0.2 to 6.9)	0.036

Baseline (Placebo: *n* = 40, Probiotic: *n* = 39), 16 weeks (Placebo: *n* = 39, *Probiotic*: *n* = 39). Values are indicated as mean (SD). Differences are indicated by changes of LSM between Placebo and *Probiotic* at 16 weeks. Effect of Probiotic was indicated in intergroup difference (95% CI) and p value by ANCOVA in per-protocol (PP) analysis. RBANS, Repeatable Battery for the Assessment of Neuropsychological Status, JMCIS, The Japanese version of the MCI Screen.

### Safety evaluation

Results of the hematological and biological blood parameters comparison between baseline and after 16 weeks of consumption did not show any significant changes. Vital signs that include blood pressure and heart rate were also unchanged. Reported adherence was 97.5% and 100% in placebo and probiotic groups, respectively. No study related adverse events occurred.

## DISCUSSION

The present study is the first double-blind, placebo-controlled study in humans to show the cognitive function enhancement benefit of the probiotic *B. breve* A1 in subjects with suspected MCI. Primary (RBANS) and secondary endpoints (JMCIS) were both met after 16 weeks of consumption in this population. The treatment was well-tolerated with no reported side-effects. We used RBANS in our study because, since its introduction in 1998, it has been proven sensitive at both detecting and characterizing very mild cognitive impairment, to distinguish dementia of different etiologies and to characterize MCI due to AD [17]. It has also been used in previous clinical studies as a sensitive measure of cognitive improvement after treatment with food products such as PUFA in the MCI population [18].

A significant improvement of cognitive functions was observed for treated participants over placebo in our study. RBANS score showed a significant 11.3-point improvement with *B. breve* A1 compared to placebo (*p* < 0.0001). RBANS domain scores were also improved: immediate memory (*p* < 0.0001), visuospatial/constructional (*p* < 0.0001), and delayed memory (*p* < 0.0001). Only language and attention domain scores saw no improvement over placebo. These data indicate that RBANS seems to be a sensitive and useful neuropsychological test to evaluate the central effects of probiotics on the memory of suspected MCI subjects and the effect of the probiotic strain in improving memory functions such as the awareness of who, when and where. The 11.3-point improvement seen after 16 weeks of *B. breve* A1 is remarkable. In comparison, dietary supplementation of arachidonic and docosahexaenoic acids in a similar MCI population showed a significant improvement of around 6 points in RBANS immediate memory score after 13 weeks [18]. Another clinical study that evaluated *Lactobacillus* fermented milk consumption effect on memory found a small but significant change of four points on the domain score of attention but no significant difference on RBANS total score [19]. Future longer longitudinal studies with *B. breve* A1 may reveal further tangible memory improvement.

A reduction of medial temporal lobe (MTL) volumes and a significantly smaller hippocampus have been previously demonstrated in MCI individuals [20]. MCI subjects also have a larger inferior lateral ventricle volume than usual. MTL volumes are significantly related to the RBANS immediate and delayed memory scores in a previous study [21], and both scores were significantly improved after *B. breve* A1 consumption. The MTL and the hippocampus are critical for short-term and long-term memory, which suggests that *B. breve* A1 is causing positive changes to the MCI subjects hippocampus, something that we also observed in our previous pre-clinical study [14]. In that study, *B. breve* A1 improved mouse memory and suppressed the hippocampal expressions of inflammation and immune-reactive genes that are induced by amyloid-*β*, and this has been reported recently also using other *Bifidobacterium* strain [22]. While we cannot investigate gene expression alteration in the hippocampus of living individuals, we speculate that *B. breve* A1 may have caused similar changes in treated study participants. Future positron emission tomography imaging studies (PET) using TranSlocatorPrOtein (TSPO), a marker of brain inflammation that was used in MCI subjects [23] to study microglial activation/inflammation, would be helpful to visualize *B. breve* A1 extent effect on the brain non-invasively. Taking into account the possibility that MCI is closely associated with the immune system and inflammation, it will be valuable in future studies to assess the effectiveness of our probiotic in MCI patients with underlying conditions that include vascular impairment or cancer.

*Bifidobacterium*, including its metabolite acetate, a short-chain fat acid, has been shown to modulate the gut microbiota and the immune system [24, 25]. In our previous pre-clinical study, we found that non-viable components of the bacterium or its metabolite acetate partially ameliorated the cognitive decline observed in an AD mouse model [14]. In that study, the administration of *B. breve* A1 increased the plasma acetate levels in treated animals. Although our present study did not compare the alteration of the gut microbiota after treatment, there is a possibility that the cognitive improvement we observed comes from a change of the gut microbiota towards less pro-inflammatory gut bacteria species, many of which are known to release lipopolysaccharides (LPS) and other metabolites leading to microglia activation in the brain [12]. Gut microbiota alteration via microbial-derived indole derivatives production is also associated with intestinal epithelial barrier integrity and modulation of intestinal inflammation [12], which may result in microglia modulation in the brain of MCI subjects after *B. breve* A1 consumption. Recently, we demonstrated that human-residential bifidobacteria, including strain *B. breve* A1, are potential producers of indole-3-lactic acid, a metabolite that has an anti-inflammatory effect and that is also possibly involved in host-microbiota crosstalk and neuronal developmental processes [26–28].

Changes in brain BDNF have been reported for probiotics such as *Bifidobacterium* in rodent experiments [29] and have a beneficial effect on genes and inflammation pathways involved in neurological disorders [30]. BDNF serum levels are elevated in MCI and AD compared to healthy individuals [31], so it is not clear if future studies could measure serum BDNF to understand how *B. breve* A1 consumption leads to memory improvement in MCI subjects. Fecal microbiota comparison after treatment in humans, as well as LPS production by the gut microbiota, will help in future studies to shed light on the mechanism of action of this probiotic strain on cognition and inflammation. This is of interest since manipulations of pre-clinical mouse models of AD using germ-free conditions and alterations of the gut microbiota with antibiotics have shown the importance of gut bacteria to influence amyloid deposition in the brain as well as stimulating microglia activation, a significant contributor to brain inflammation and cognitive impairment in MCI [32].

In our next clinical trials, we will conduct exploratory biomarker studies using blood, cerebrospinal fluid, and feces to fully understand how *B. breve* A1 is causing an amelioration of memory in the MCI population as well as evaluate its potential to treat AD dementia. The identification of the precise mechanism would also shed much-needed light on what is causing dementia and related CNS disorders and potentially help justify further intervention studies in various neuropathologies.

No drugs are currently approved for treating MCI, as opposed to dementia. Approved symptomatic drugs for AD such as donepezil, rivastigmine, and galantamine were thought to potentially help with symptoms of MCI, or slow its progression to dementia, but clinical trials data turned negative. The identification of effective treatment of MCI subjects is, therefore, a pressing unmet medical need. In our study, *B. breve* A1 showed a clear and significant improvement of RBANS total score, in particular for domain scores of immediate memory, visuospatial/constructional and delayed memory, and JMCIS after only 16 weeks in the suspected MCI population. This finding, although seeking subsequent confirming studies, could signal a profound shift as to how MCI can be treated and perhaps, as a result, prevent the development of cognitive impairment by an affordable and safe solution to the general population.
